# Prediction of cardiovascular disease risk using waist circumference among Aboriginals in a remote Australian community

**DOI:** 10.1186/s12889-015-1406-1

**Published:** 2015-01-31

**Authors:** Odewumi Adegbija, Wendy Hoy, Zhiqiang Wang

**Affiliations:** Centre for Chronic Disease, School of Medicine, University of Queensland, Brisbane, Australia

**Keywords:** Waist circumference (WC), Cardiovascular disease (CVD), Hazard ratio, Absolute risk

## Abstract

**Background:**

Elevated waist circumference (WC) is an important risk factor for cardiovascular disease (CVD). Aboriginals in Australia are at higher risk of CVD compared to non-Aboriginals. We examined the association between waist circumference and CVD, and developed a model for projecting absolute risk of cardiovascular disease using WC and age in one high risk Australian Aboriginal community.

**Methods:**

We followed up 920 (470 men, 450 women) participants (more than 80% of the eligible population) aged 18 to 76 years, without CVD at baseline, for up to 20 years. Hazard ratios were estimated using Cox proportional hazards models adjusting for potential confounding factors. Absolute risk was estimated using the Weibull regression model.

**Results:**

Of 920 study participants, 156 males and 177 females developed CVD in the follow-up period. Incidence rates for males and females in the 4th WC quartile (Q4) were 38.3 (95% CI 29.6, 49.7) and 47.2 (95% CI 37.1, 60.3) respectively. Crude hazard ratios of CVD for Q4 WC group using Q1 (quartile 1) as the referent quartile were 2.9 (95% CI 1.8- 4.6) for males and 3.5 (95% CI 2.2- 5.5) for females. Association remained after controlling for age, smoking status and alcohol drinking status (HR = 1.8 for males and HR = 3.1 for females). At 45 years of age with baseline waist circumference of 100 cm, a male had an absolute CVD risk of 32.5%, while a female had a 30.6% risk of the disease.

**Conclusions:**

Risk of CVD among participants increased with increasing WC, and the relationship was accentuated with increasing age. The prediction model provides a tool for understanding the combined effects of WC with age on CVD events in the Australian Aboriginal community. It is simple and easily understood and will assist in identifying individuals at risk of CVD in relation to waist circumference values.

## Background

Cardiovascular disease (CVD) is the second largest contributor to the total disease burden in Australia, accounting for about 16% of the total disease burden and recorded as the underlying cause of 46,100 and 45,600 deaths in 2009 and 2011 respectively [1-3]. Inequality exists in the number of those affected by CVD with greater impact on the Aboriginals than non-Aboriginals in Australian [2,4,5]. Among the Australian Aboriginal population, CVD is the leading cause of disease burden and deaths and lists as one of four chronic conditions that accounts for 70% of the indigenous Australian health gap [1]. Australian hospitalization records for CVD were reported at about 1.7 times among Aboriginals in comparison to other Australians [4]. As CVD is a substantially significant contributor to illnesses, disability and premature death particularly among the Aboriginal group [4,6], there is a need to alert them of health risks associated with greater CVD risk among them.

In the management of CVD, guidelines have been provided to predict CVD in the presence of a number of the modifiable and non-modifiable risk factors. However, most of the available guidelines are based on non-specific populations resulting in unsuitability of the tool for some groups of individuals. Some modifiable risk factors identified with CVD include overweight and obesity, tobacco smoking, diabetes, unhealthy diet, high blood pressure, high blood cholesterol and physical inactivity. Incidence of CVD also increases with non-modifiable risk factors such as age and ethnicity. Australia’s cardiovascular risk chart related an individual’s diabetes status, sex, smoking history and age with systolic blood pressure and cholesterol levels to present the risk of CVD [7]. However, the CVD risk chart focused on the non-Aboriginal Australian population while also targeting middle aged Aboriginals (35 to 44 years of age) only, without considering differences in body habitus of Australians. Aboriginal Australians vary in their body habitus which includes waist circumference (WC) compared to non- Aboriginal Australians [8-11]. This Australian group also have the tendency of abdominal fat storage, particularly in women, having relatively large WC measurements [9,12]. The phenomenon of higher waist circumference in Aboriginal females compared to males, have been reviewed in a meta-analysis conducted to compare WC estimates in Aboriginal and non-Aboriginal Australian populations [13]. Some studies in Australia that examined the relationship between WC and risk of CVD included other anthropometric indices such as body mass index (BMI) and waist-to-hip ratio (WHR) to present the best predictor of the disease and the findings have been controversial. While WHR compared to WC and BMI was found to be the most useful obesity measure in identifying individuals with CVD risk among non-Aboriginal Australians [14], WC better predicted CVD compared to BMI and WHR among Aboriginals [15,16]. Due to the differences in WC profiles and the higher level of CVD risk of Aboriginals compared to non-Aboriginals in Australia, this study evaluated the relationship between WC and risk of developing CVD during the 20 years of follow-up study period. Also, we developed a simplified cardiovascular prediction model by estimation of absolute risk of CVD using different waist circumference (WC) values, over a 10 year period in Aboriginal subjects between the ages of 20 to 65 years in a remote Australian community. This prediction tool can be used to educate and alert Aboriginals of the risk of developing CVD according to WC and age. Furthermore, this tool will be helpful for the planning and conducting obesity-related health education programs for the prevention and management of CVD in Aboriginal communities in Australia.

## Methods

### Study design

This was a prospective cohort study in a remote Aboriginal community in Australia designed to follow up adult individuals with WC measurement at baseline for up to 20 years to identify newly-diagnosed CVD events.

### Study population

The original cohort included 1490 participants from a remote Aboriginal community in Australian’s Northern Territory (NT), whose baseline data were collected from 1992. Those participants were followed for up to 20 years until 31st of May 2012.

Eligible participants recruited from the study group fulfilled the following criteria: 1) aged between 18 and 76 years, 2) had WC measurement at baseline and 3) free from known CVD at baseline. A total of 920 (470 men and 450 women) participants met the study criteria. CVD events were defined according to the *English Revision International Classification of Diseases (*ICD-9 code) *codes 390 to 458* and *International Statistical Classification of Diseases, 10th Revision (*ICD-10 code*) codes I00 to I99*. We eliminated those with hypo- and hypertensive diseases and haemorrhoids as these occurrences may be physiologic (as with hypotension) rather than a disease, or with no obvious underlying medical cause (primary hypertension). Each study participant had been assigned a unique Study Identification Number (SIN) to merge the baseline, follow-up morbidity data sets and the mortality data (death records helped identify those who had died as a result of CVD but had not been recorded in the hospital records, to add to our list of those who developed the disease). Our end point was incidence of CVD among study participants. Therefore, participants were followed up to identify cases of newly diagnosed CVD. Written informed consents were obtained from all participants at baseline measurements. The original baseline data collection was approved by the relevant Aboriginal community and the Menzies School of Health Research Ethics Committees and the hospitalization data collection for the current project was approved by the University of Queensland.

### Baseline measurements

Existing baseline dataset was used for the analysis of this study and has been described elsewhere [17,18]. In summary, characteristics of participants were collected through interview questionnaires and they included demographic and clinical variables such as age, gender, WC, height, weight, waist-to-hip ratio, smoking status and alcohol consumption. Waist circumference was measured using flexible tapes on a horizontal plane, midway between the lower border of the ribs and the iliac crest. Height and weight were measured with subjects wearing light clothing and no shoes and body mass index (BMI) was calculated as weight in kilograms divided by height in meters squared. Due to the disparity in WC of Australian Aboriginals and non-Aboriginals, the currently recommended WC cut-off points which were developed for individuals of European origin might not be applicable to this population [19]. Therefore, for the current study, waist circumference was grouped into gender-specific quartiles. Quartiles for males (Q1 = 63–78 cm, Q2 = 79–85 cm, Q3 = 86–95 cm, Q4 = 96–138 cm). Quartiles for females (Q1 = 60–79 cm, Q2 = 80–90 cm, Q3 = 91–101 cm, Q4 = 101.5- 135 cm). Q1 was the reference group for comparison.

### Statistical analysis

Continuous variables were expressed as median and interquartile range (IQR). We expressed categorical variables as frequencies and percentages. Using the Cox proportional hazards model, we assessed the association between WC and CVD outcomes adjusting for potential confounding factors (age, smoking status and alcohol consumption status). Age was a continuous variable, while smoking (smokers and non-smokers) and alcohol consumption (drinkers and non-drinkers) were categorical variables. Separate hazard functions were calculated for males and females. Survival was calculated using the Kaplan Meier proportional survival probability estimates. To predict 10-year risk of CVD using WC and age as covariates, we treated both age and WC as continuous variables. We used the Weibull regression to estimate the absolute risk of developing CVD. To estimate survival S(t_j_|X_j_) = exp[−{exp(−β_0_- X_j_ β_j_)t_j_}^p^], we estimated parameters β_0_, β_j_ and p; where β_0_ represented the baseline coefficient, β_j_ was the regression coefficient for covariates (WC and age), X_j_ represented the value of the covariates (WC and age), t = time and p = the shape parameter that indicates the hazard level. To compare the associations of WC, BMI and WHR with CVD between males and females, we converted original WC, BMI and WHR values into gender specific z-scores for both genders while also controlling for age, smoking and alcohol consumption status. Data were analyzed using Stata 12 [20]. A P-value of <0.05 was considered to be statistically significant.

## Results

### Characteristics of study participants

The baseline characteristics for 920 study participants who met the study criteria are presented in Table 1. The median (IQR) ages at baseline screening for men and women were 31 (15) years and 34 (20) years respectively. Waist circumference median (IQR) for men was 86 (17) cm and 91 (22) cm for women. BMI median was slightly higher in males than in females (22.9 kg/m^2^ versus 23.9 kg/m^2^). The median (IQR) WHR for males and females were 1.0 (0.1) and 0.9 (0.1) respectively. Men had higher median body weight estimates compared to women (67.5 kg versus 61.0 kg). 373 (80%) males and 296 (67%) of females were smokers. 394 (85%) males and 153 (35%) females were alcohol consumers from the total population.

**Table 1:** **Baseline descriptive statistics and clinical data of male and female participants**

	**Males**	**Females**
**Demographics**		
Age, years- median (IQR)	31 (15)	34 (20)
Gender (n, %)	470 (51.1)	450 (48.91)
**Physical findings – median (IQR)**		
Waist circumference (cm)	86(17)	91 (22)
BMI (kg/m^2^)	22.9 (6.3)	23.9 (8.6)
Waist-to-hip ratio	1.0 (0.1)	0.9 (0.1)
Weight (kg)	67.5 (19)	61 (24)
Height (cm)	172 (8.5)	161 (8.0)
**Lifestyle factors- n (%)**		
Current smoker	373 (80.0)	296 (67.0)
Current alcohol drinker	394 (84.6)	153 (34.7)

IQR: Interquartile range.

During the 20 year follow-up period, 333 (36.2%) people- 156 men and 177 women were diagnosed as having cardiovascular disease. A total of 87.3% of the cohort were observed for at least five years, and 1.6% was observed for one year or less. 24.3% of the CVD events have occurred within the first 5 years of follow- up in 35 men and 46 women, and the proportion increased to 50.5% within 10 years of follow-up with a further increase to 82.0% at 15 years follow-up.

### Incidence rates of CVD

**Figure 1:**
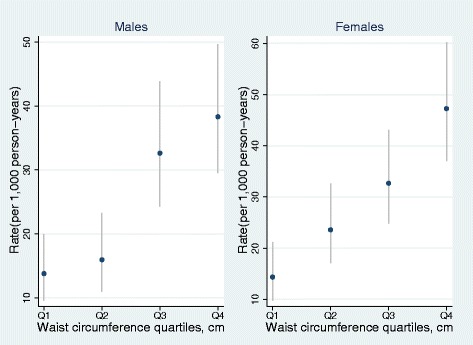
**Rate per 1,000 person-years of males and females by WC quartiles measured in cm.**

The incidence rate of developing hospital diagnosed CVD was 23.8/ 1,000 person-years for males while the incidence rate for females was 28.5/ 1,000 person. Because of the differences in WC of males and females, waist circumference was categorized into gender-specific quartiles. Table 2 shows numbers of CVD cases and incidence rates by WC quartiles. Rates ranged from 13.8 (95% CI 9.5, 20.0) in Q1 WC quartile to 38.3 (95% CI 29.6, 49.7) in Q4 for males and 14.3 (95% CI 9.7, 21.2) in Q1 to 47.2 (95% CI 37.1, 60.3) in Q4 for females. As shown in Figure 1, CVD incidence rate among participants increased considerably with increasing WC and females had higher rates than males in the 4th WC quartile.

**Table 2:** **WC quartiles and rate per 1,000 person-years (95% confidence intervals) of participants during the 20-year follow-up**

	**Males**			**Females**		
**Waist quartiles(cm)**	**CVD cases**	**Person-years**	**Rate (95% CI)**	**CVD cases**	**Person-years**	**Rate (95% CI)**
Q1	28	2029.0	13.8 (9.5, 20.0)	25	1748.3	14.3 (9.7, 21.2)
Q2	27	1687.5	16.0 (10.9, 23.3)	37	1567.8	23.6 (17.1, 32.6)
Q3	44	1349.7	32.6 (24.3, 43.9)	50	1529.1	32.7 (24.8, 43.1)
Q4	57	1488.3	38.3 (29.6, 49.7)	65	1377.1	47.2 (37.1, 60.3)

Quartiles for males (Q1 = 63–78 cm, Q2 = 79–85 cm, Q3 = 86–95 cm, Q4 = 96–138 cm).

Quartiles for females (Q1 = 60–79 cm, Q2 = 80–90 cm, Q3 = 91–101 cm, Q4 = 101.5- 135 cm).

### Association between WC and CVD

Table 3 shows the hazard ratios of CVD for different WC quartiles. The crude association between WC and CVD were statistically significant for both genders. In comparison to the referent WC quartile (Q1), crude HR for males in Q4 was 2.9 (95% CI: 1.8- 4.6), while HR for females in Q4 was 3.5 (95% CI: 2.2- 5.5). After adjusting for age, smoking status and alcohol status, association between WC and CVD remained strong for both males and females. When WC and age were included in the same multivariate model (results not shown in Tables), the HRs for Q2, Q3, Q4 in comparison to Q1 were 0.9, 1.6 and 1,6 respectively for men and 1.5, 1.9 and 2.9 accordingly for women. The model that included WC and smoking gave HRs for Q2 = 1.2, Q3 = 2.5 and Q4 = 3.0 for males and Q2 = 1.8, Q3 = 2.5 and Q4 = 3.6 for females. Multivariate model including WC and alcohol consumption were same as WC and smoking for both males and females. To assess the gender effect using WC quartiles, HR = 1.1 (95% CI: 0.9- 1.4) showing women had slightly higher risk of CVD than men. However, there were no significant differences between males and females, P-value = 0.4.

**Table 3:** **Crude and adjusted association between WC and CVD**

	**Crude**		*Adjusted	
**Males**	**HR (95% CI)**	**P-value**	**HR (95% CI)**	**P-value**
WC quartiles				
Q1	1	<0.0001	1	<0.0001
Q2	1.2 (0.7, 2.0)		0.9 (0.5, 1.5)	
Q3	2.5 (1.5, 4.0)		1.6 (1.0, 2.7)	
Q4	2.9 (1.8, 4.6)		1.8 (1.1, 2.9)	
**Females**				
WC quartiles				
Q1	1	<0.0001	1	<0.0001
Q2	1.7 (1.0, 2.8)		1.7 (1.0, 2.8)	
Q3	2.4 (1.5, 3.8)		2.1 (1.3, 3.5)	
Q4	3.5 (2.2, 5.5)		3.1 (1.9, 5.0)	

*Adjusted for age, smoking status and alcohol status.

HR: Hazard ratio; CI: confidence intervals.

Quartiles for males (Q1 = 63–78 cm, Q2 = 79–85 cm, Q3 = 86–95 cm, Q4 = 96–138 cm).

Quartiles for females (Q1 = 60–79 cm, Q2 = 80–90 cm, Q3 = 91–101 cm, Q4 = 101.5- 135 cm).

WC was a better predictor of CVD than body mass index (BMI) and waist-to-hip ratio (WHR) particularly among women as shown in Table 4. We converted original WC, BMI and WHR values into standard deviation scores (z-score) to make their associations with CVD comparable. For males, 1 standard deviation (SD) increase in WC, BMI and WHR increased CVD risk by 1.6 (95% CI: 1.4, 1.8), 1.3 (95% CI: 1.2, 1.6) and 1.6 (95% CI: 1.4- 1.8) respectively. For females, 1 SD increase in WC, BMI and WHR increased CVD risk by 1.5 (95% CI: 1.3, 1.8), 1.3 (95% CI: 1.2, 1.6) and 1.3 (95% CI: 1.1- 1.5) respectively. When WC, BMI and WHR were independently included in models containing other risk factors of CVD (age, cigarette smoking and alcohol consumption status) to assess association with CVD, statistical significance was observed in the results of both genders.

**Table 4:** **Hazard ratios of z-scores of crude and adjusted estimates of WC, BMI and WHR**

**Variable**	**Crude**		*Adjusted	
	**HR (95% CI)**	**P-value**	**HR (95% CI)**	**P-value**
Waist Circumference (cm)				
Males	1.6 (1.4-1.8)	<0.0001	1.4 (1.2-1.7)	<0.0001
Females	1.5 (1.3- 1.8)	<0.0001	1.4 (1.2- 1.7)	<0.0001
Body mass index (Kg/m^2^)				
Males	1.3 (1.2- 1.6)	<0.0001	1.2 (1.0- 1.5)	0.01
Females	1.3 (1.2- 1.6)	<0.0001	1.3(1.1- 1.5)	<0.0001
Waist-to-hip ratio				
Males	1.6 (1.4- 1.8)	<0.0001	1.4 (1.2- 1.7)	<0.0001
Females	1.3 (1.1- 1.5)	<0.0001	1.2 (1.1- 1.4)	<0.0001

*Adjusted for age, smoking status and alcohol status.

HR (Hazard ratio); 95% CI (95% confidence interval).

### Absolute risk of CVD

Absolute risk (Males) = 1 – (exp[−{exp(−5.9099- (−0.0157*WC – 0.0305*Age))*t_j_}^1.4143^]),

Absolute risk (Females) = 1 – (exp[−{exp(−5.6569- (−0.0174*WC −0.0198*Age))*t_j_}^1.3965^]).

**Figure 2:**
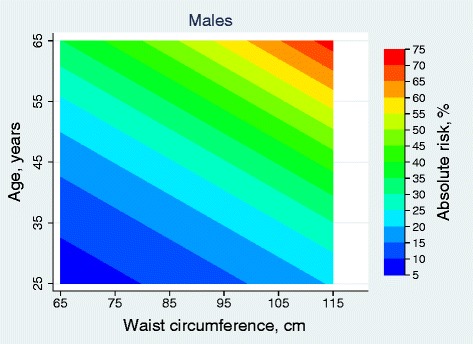
**Absolute 10-year risk (%) of cardiovascular disease for males, using waist circumference (cm) and age (years).**

**Figure 3:**
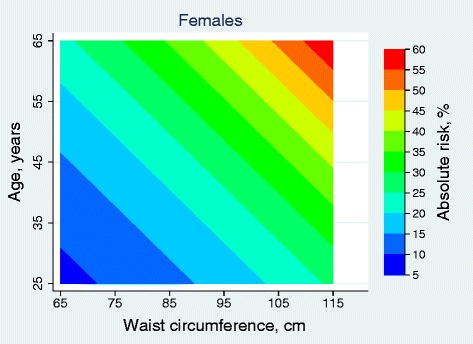
**Absolute 10-year risk (%) of cardiovascular disease for females, using waist circumference (cm) and age (years).**

Based on the coefficients of the final Weibull models above, we estimated 10-year absolute risks according to age and WC values. The predicted risk estimates (shown as percentages) for 10-year CVD were calculated and presented in Figures 2 and 3. The risk of CVD was driven by both age and WC values and it increased with higher WC values and increasing age.

The use of Figures 2 and 3 to make CVD predictions is best illustrated by example. Specifically, individual men less than 32 years of age with WC < 80 cm had a 10 year absolute CVD risk of 5- 10%; those between 42 and 49 years with WC range of 100–112 cm had a 10 year absolute risk of 15- 20%; and those above 50 years and WC above 90 cm had 10 year absolute risk of above 50%.

For women, participants less than 30 years with WC less than 70 cm had a 10 year absolute CVD risk of 5- 10%; individuals between 48 and 57 years with WC between 90 and 100 cm had an absolute risk of 15- 20%; and above 57 years and WC above 105 cm had 10 year absolute risk above 50%.

Gender effect for the same absolute WC risk was not significant HR = 1.0 (95% CI: 0.8- 1.3, p = 0.9), indicating males and females were not different in their risk of CVD given a WC value.

**Figure 4:**
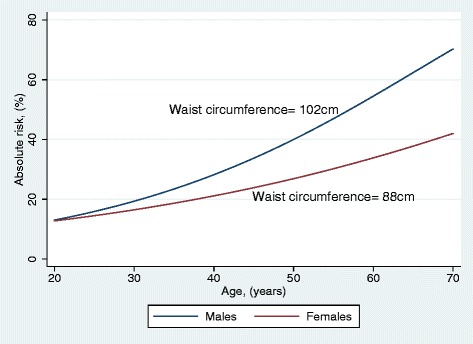
**Absolute risks (%) of CVD for males and females with currently established WC cut-off values for obesity.**

We next examined the absolute risk of CVD using age and the currently recommended WC-cut-off values for obesity (102 cm for males and 88 cm for females) [19]. As shown in Figure 4, at the age of 30 years, absolute risk of CVD was less than 20% for obese individuals. Obese males and females aged 50 years had an absolute CVD risk of about 40% and 26% respectively. Absolute CVD risk was higher in males than females with these WC cut-off values.

## Discussion

In the present study, we followed up Aboriginal Australians participating in a baseline community screening program for a maximum of 20 years for first episode of cardiovascular disease. Females in this study had higher WC estimates in comparison to the males. On the other hand, men were taller, had higher weight estimates, and were twice more likely alcohol drinkers and 1.2 times likely smokers when compared to the women. There were no distinctive gender differences in CVD risk for a particular WC value. WC measured at baseline predicted the risk of CVD as the risk increased with increasing WC, with the highest risk in the 4th WC quartile for both genders. The hazard ratio of WC was attenuated with the adjustment of age in the same multivariate model. However, adjusting for smoking and alcohol made little or no difference on the association between WC and the risk of CVD in the model. WC compared to BMI and WHR was a better predictor of CVD particularly in women. Finally, the absolute risks of CVD increased with increasing WC values and age in this population. The availability of this simple tool to predict future risk of CVD should improve the understanding of WC on CVD risk and identify high-risk individuals based on WC and age. It can also serve as an adjunct for planning and conducting public health education programs for CVD and augment preventive strategies for Aboriginal communities.

The scarcity of reports on absolute risk of CVD and risk factors such as WC and age in indigenous populations of Australia and other countries makes it difficult to compare our findings on absolute risks of CVD morbidity and WC. Nonetheless, our results are in general consistent with the findings from other ethnic populations on the risk associated with high WC and development of CVD [21-23]. Hans et al. (1995) used the currently recommended WC-cut off points to assess differences in the risk of CVD among overweight and obese individuals, and reported individuals with WC in the obese group had higher CVD risk compared to those in the overweight category [23]. Likewise, two studies in the United States used the Physicians’ Health Study for men [21] and the Nurses’ health Study cohort for women [22] to assess abdominal adiposity and coronary heart disease (CHD) and found that the highest WC quintile had higher risk of developing CHD than those in the lowest quintile. In our study, risk of developing CVD increased as WC increased with the highest risk in the 4th WC quartile. Although, females in the highest WC quartile had higher risk than their male counterparts (from results of hazard ratios in Table 3), the gender effect was not statistically significant. This effect could potentially be due to small sample size or some other possible reasons which we cannot explain.

The uniqueness of the present study is reflected in the absolute risk graphs presented to provide a general overview of the combined effects of WC and age on CVD in the Aboriginal community. The currently used Australian CVD risk chart targeted the Australian population while combining the effects of some risk factors without considering WC which is strongly associated with risk of CVD among Aboriginals [15]. Importantly, our study targeted a specific Aboriginal community where WC was strongly associated with risk of CVD [15]. Also, we included age in the absolute risk prediction due to the association of increasing age with CVD risk [24]. The effect combination of WC and age to predict absolute risk of CVD in this community would assist in efficiently identifying unrecognized CVD in this community. Furthermore, this study shows how absolute risk of CVD changes with WC values as age increases. We adjusted for smoking in our analysis as it has been found to be a CVD risk factor that affects body weight [25,26]. Our findings showed men tend to develop CVD at a younger age than women while the reverse was apparent for females who were more likely to develop CVD with greater WC than increasing age. This is understandable as WC of Australian Aboriginal women have been shown to have higher estimates than that of men.

The currently recommended WC cut-off values for obesity, 102 cm for men and 88 cm for women [27] contradict our findings. Applying these cut-off values to Aboriginal men and women, the absolute risks were much higher for males than females (Figure 4). Risk of developing CVD increased to 70% and 41% among obese males and females respectively. As shown by our analyses in the incidence rates and hazard ratio estimates, females showed higher risk particularly in the highest WC quartile indicating higher risk of developing the disease among females than males with high WC, albeit, males and females were no different in their CVD risk with same absolute WC values. The currently used WC cut-offs either gives an underestimation of risk in women or an elevated risk in men in this population. Therefore, these WC cut-offs might not be applicable to this Aboriginal community. Adjusting the WC cut-off values to suit Aboriginal men and women will be beneficial to make their risks of CVD comparable.

There may be questions around the accuracy of prediction attained with using only WC and age rather than a combination of factors as seen in previous studies [28,29]. The purpose of this study was to examine the relationship between WC and CVD in relation to age. Moreover, our models based on the current data suggest that WC could be a better predictor than BMI and WHR in females for developing hospital diagnosed CVD in this population [15]. On the other hand, for males, our findings concur with a previous Canadian study in concluding that WC was a better predictor of CVD than BMI but not WHR [30]. Our results on WC as the best measure compared to WHR and/or BMI in identifying individuals with CVD risk are in agreement with earlier studies [30,31]. Dalton et al. (2003), in a previous Australian national study presented a contrasting result to ours when they demonstrated WHR was a more useful obesity measure than WC and BMI to identify individuals with CVD [14]. However, they focused on non-Aboriginals and used a cross-sectional study design which might not present the causal association between the measures and CVD. While we are not comparing WC, BMI and WHR in CVD risk prediction models in presenting a more accurate estimate, emphasis is being placed on the significant role that increasing WC plays in the development of CVD. Importantly, this is the first study assessing the role of WC to the CVD absolute risk prediction in relation to age in this population.

### Strengths and limitations

To the best of our knowledge, this is the first CVD prediction model that will include waist circumference in an indigenous Australian population. Our study is population-based comprising of individuals from a homogenous population with over 80% of the eligible community members involved in the baseline screening survey. The prospective design and high rate of follow-up (up to 20 years) minimized the potential for recall bias and loss to follow-up enabling the capture of a good number of CVD morbidity events in the community. Reverse causality bias is unlikely in our study since we excluded those with existing CVD at baseline and included only the first episode of CVD after baseline measurement.

This study may be limited by WC measurements which were from routine examinations and were subject to measurement errors. Second, our end-point was limited as we excluded hyper- and hypotensive as well as haemorrhoids cases. However, we did a cross check to ensure that those with first episodes of the excluded events were free of CVD till end of the follow-up period; otherwise those who developed the disease were included in the analysis. Third, potential confounding effects of other variables (diet, physical activities and family history of CVD) were not adjusted for in the reported strong association between WC and CVD risk in this study as we do not have data on these important risk factors. Therefore, we could only analyze on data available to us (age, WC, smoking, gender, alcohol consumption) in our analysis. Fourth, there may be questions around the use of baseline WC measurement in the prediction of future CVD events without considering the changes in WC over the follow-up time. This is unlikely to affect our findings as the purpose is to relate WC measured at baseline with when CVD develops in the follow-up time. Moreover, WC estimates are not static but change with time depending on the age and lifestyle of individuals. Lastly, findings of this study may not be generalizable to other Aboriginal populations as results were based on just one Aboriginal community. As heterogeneity of body habitus profiles which includes WC vary in different Aboriginal communities, further research will be required to collect data from other Aboriginal communities and analysis carried out to assess whether results are comparable to the present study.

## Conclusion

Our results suggest that WC may be a valuable factor in the CVD risk prediction models particularly in Australian indigenous communities whose people have higher propensity for abdominal fat than the general Australians. Although the gender effect was quite small for same absolute WC values in favor of females, the differences were not significant between males and females. Our findings suggest that currently used WC cut-off values in Australia will capture different absolute risks of CVD in Aboriginal men and women and may need to be re-evaluated; a lower cut-off for males and higher cut-off for females may be more appropriate for better reflection of risk of CVD. The findings of this study can be used by health professionals to communicate knowledge of the risk of high WC estimates to individuals in the community. This tool could serve to raise awareness of CVD among the people due to the significant burden of illnesses and premature deaths in the indigenous population.

## Abbreviations

WC: Waist circumference
BMI: Body mass index
WHR: Waist-to-hip ratio
cm: centimetres
CVD: Cardiovascular diseases
Quartile 1: Q1, Quartile 2: Q2, Quartile 3: Q3, Quartile 4: Q4

## References

[1] Australian Institute of Health and Welfare, Australia’s Health 2010 (2010). The twelfth health report of the Australian Institute of Health and Welfare. Cat. No. AUS 122.

[2] Australian Institute of Health and Welfare, Australia’s Health (2012). The thirteenth biennial health report of the Australian Institute of Health and Welfare. Cat. No. AUS 156.

[3] National Health and Medical Research Council: Cardiovascular Disease (National Health Priorities Areas). [https://www.nhmrc.gov.au/grants-funding/research-funding-statistics-and-data/funding-statistics-grants-and-funding-data/cardi]

[4] Australian Institute of Health and Welfare. Cardiovascular Disease. Australian facts 2011. 2011; p. 1–232.

[5] Gray C, Brown A, Thomson N (2012). Review of cardiovascular health among Indigenous Australians. Australian Indigenous HealthInfoNet.

[6] Heart Foundation. The shifting burden of cardiovascular disease in Australia. Access Economics, 2005: p. 1–103.

[7] Heart Foundation. Absolute cardiovascular disease risk assessment. Heart Foundation Guide to management of hypertension. 2008: p. 1–6.

[8] Li M, McDermott RA (2010). Using anthropometric indices to predict cardio-metabolic risk factors in Australian indigenous populations. Diabetes Res Clin Pract.

[9] Kondalsamy-Chennakesavan S, Hoy WE, Wang Z, Briganti E, Polkinghorne K, Chadban S (2008). Anthropometric measurements of Australian Aboriginal adults living in remote areas: comparison with nationally representative findings. Am J Hum Biol.

[10] Wang Z, Hoy WE (2003). Hypertension, dyslipidemia, body mass index, diabetes and smoking status in Aboriginal Australians in a remote community. Ethn Dis.

[11] Li M, McCulloch B, McDermott R (2012). Metabolic syndrome and incident coronary heart disease in Australian indigenous populations. Obesity (Silver Spring).

[12] O’Dea K (1987). Body fat distribution and health outcome in Australian Aborigines. Proc Nutr Soc Aust.

[13] Adegbija OO, Wang Z (2014). Gender variations in waist circumference levels between aboriginal and non-aboriginal Australian populations: a systematic review. Obesity Research and Clinical Practice.

[14] Dalton M, Cameron AJ, Zimmet PZ, Shaw JE, Jolley D, Dunstan DW (2003). Waist circumference, wait-to hip ratio and body mass index and their correlation with cardiovascular disease risk factors in Australian adults. J Intern Med.

[15] Wang Z, Hoy WE (2004). Waist circumference, body mass index, hip circumference and waist-to-hip ratio as predictors of cardiovascular disease in Aboriginal people. Eur J Clin Nutr.

[16] Chan LC, Ware RS, Kesting J, Marczak M, Good D, Shaw JT (2007). Association between anthropometric measures of obesity and cardiovascular risk markers in a self-selected group of indigenous Australians. Eur J Cardiovasc Prev Rehabil.

[17] Hoy WE, Baker PR, Kelly AM, Wang Z (2000). Reducing premature death and renal failure in Australian aboriginals. A community-based cardiovascular and renal protective program. Med J Aust.

[18] Wang Z, Hoy WE (2004). Body size measurements as predictors of type 2 diabetes in Aboriginal people. Int J Obes Relat Metab Disord.

[19] Lear SA, Humphries KH, Birmingham CL (2007). The use of BMI and waist circumference as surrogates of body fat differs by ethnicity. Obesity (Silver Spring).

[20] StataCorp (2011). Stata Statistical Software: Release 12.

[21] Rexrode KM, Buring JE, Manson JE (2001). Abdominal and total adiposity and risk of coronary heart disease in men. Int J of Obes Relat Metab Disord.

[22] Rexrode KM, Carey VJ, Hennekens CH, Walters EE, Colditz GA, Stampfer MJ (1998). Abdominal Adiposity and coronary heart disease in women. JAMA.

[23] Han TS, van Leer EM, Seidell JC, Lean ME (1995). Waist circumference action levels in the identification of cardiovascular risk factors: prevalence study in a random sample. BMJ.

[24] World Heart Foundation: Cardiovascular disease risk factors. 2014; p. 1–4. [http://www.world-heart-federation.org/press/fact-sheets/cardiovascular-disease-risk-factors/]

[25] Bamia C, Trichopoulou A, Lenas D, Trichopoulous D (2004). Tobacco smoking in relation to body fat mass and distribution in a general population sample. Int J Obes Relat Metab Disord.

[26] Barrett-Connor E, Khaw KT (1989). Cigarette smoking and increased central adiposity. Ann Intern Med.

[27] Lean ME, Hans TS, Morrison CE (1995). Waist circumference as a measure for indicating need for weight management. Br Med J.

[28] D’Agostino RB, Grundy S, Sullivan LM, Wilson P (2001). Validation of the Framingham coronary heart disease prediction scores: results of a multiple ethnic groups investigation. JAMA.

[29] Conroy RM, Pyorala K, Fitzgerald AP, Sans S, Menotti A, De Backer G (2003). Estimation of ten-year risk of fatal cardiovascular disease in Europe: the SCORE project. Eur Heart J.

[30] Dobbelsteyn CJ, Joffres MR, MacLean DR, Flowerdew G (2001). The comparative evaluation of waist circumference, waist-to hip ratio and body mass index as indicators of cardiovascular risk factors. The Canadian Heart Health Surveys. Int J Obes Relat Metab Disord.

[31] Pouliot MC, Despres JP, Lemieux S, Moorjani S, Bouchard C, Tremblay A (1994). Waist circumference and abdominal sagittal diameter: best simple anthropometric indexes of abdominal visceral adipose tissue accumulation and related cardiovascular risk in men and women. Am J Cardiol.

